# Ethnopharmacological Potential of *Aspilia africana* for the Treatment of Inflammatory Diseases

**DOI:** 10.1155/2020/8091047

**Published:** 2020-06-19

**Authors:** Denis Okello, Jun Lee, Youngmin Kang

**Affiliations:** ^1^Korean Convergence Medicine Major, University of Science and Technology (UST), Daejeon, Republic of Korea; ^2^Gombe Secondary School, P.O. Box, 192, Butambala, Mpigi, Uganda; ^3^Herbal Medicine Resources Research Center, Korea Institute of Oriental Medicine, 111 Geonjae-ro, Naju-si 58245, Jeollanam-do, Republic of Korea

## Abstract

Inflammatory diseases are major health concerns affecting millions of people worldwide. *Aspilia africana* has been used for centuries by many African communities in the treatment of a wide range of health conditions, including inflammatory diseases, osteoporosis, rheumatic pains, and wounds. Analysis of the phytochemical composition of *A*. *africana* indicated that the plant is rich in a broad range of secondary metabolites, including flavonoids, alkaloids, tannins, saponins, terpenoids, sterols, phenolic compounds, and glycosides. This explains the efficacy of the plant in treating inflammation-related diseases, as well as several other health conditions affecting different African communities. The mechanisms of action of the anti-inflammatory phytochemical compounds in *A*. *africana* include inhibition of a number of physiological processes involved in the inflammatory process and synthesis or action of proinflammatory enzymes. The phytochemicals enhance anti-inflammatory biological responses such as inhibition of a number of chemical mediators including histamine, prostanoids and kinins, 5-lipoxygenase. and cyclooxygenase and activation of phosphodiesterase and transcriptase. Currently used anti-inflammatory medications are associated with several disadvantages such as drug toxicity and iatrogenic reactions, thereby complicating the treatment process. The adverse effects related to the use of these conventional synthetic drugs have been the driving force behind consideration of natural remedies, and efforts are being made toward the development of anti-inflammatory agents based on natural extracts. *A*. *africana* is rich in secondary metabolites, and its use as a traditional medicine for treating inflammatory diseases has been validated through *in vitro* and *in vivo* studies. Therefore, the plant could be further explored for potential development of novel anti-inflammatory therapeutics.

## 1. Introduction

Inflammation is a natural biological and complex reaction, typically of mammals, to a wide range of belligerent agents such as pathogens, parasites, harmful chemicals, and physical tissue injury [1]. Inflammatory response is a protective effort by the bodies of organisms to eliminate harmful stimuli and initiate the healing process in damaged tissues [2]. During inflammation, the body institutes a biochemical cascade that prompts responses geared toward healing the damaged tissue. Leucocytes become chemotactically activated, migrate toward the damaged tissue, and produce cytokines responsible for the induction of inflammatory responses [3]. Various cytokines and cells are involved in the early stages of defense, in an attempt to restrict tissue damage to the affected area [4]. Inflammation at the tissue level is characterized by certain events that include pain, swelling, redness, heat, and loss of function caused by local immune and vascular cell responses to injury or infection [3, 5]. It is considered one of the main causes for the development of a number of diseases such as cancer, inflammatory bowel disease, diabetes, osteoporosis, cardiovascular disease, asthma, rheumatoid arthritis, as well as central nervous system disorders, including Alzheimer's disease, Parkinson's disease, and depression [4, 5]. In fact, inflammatory diseases are a major global health concern affecting millions of people and have a significant socioeconomic impact aside from being one of the most relevant areas of medical research [6].

*A*. *africana* (Pers.) C. D. Adams belongs to the family Asteraceae and has been used by many African communities in the treatment of a range of health conditions. The plant is used to treat inflammatory conditions as well as osteoporosis, stomach ache, diarrhea, measles, malaria, tuberculosis, cough, gastric ulcers, sores, diabetes, rheumatic pains, bee, scorpion and wasp stings, ear infections, febrile headaches, and gonorrhea and is used as a contraceptive. *A*. *africana* is also prominently known for its wound healing properties [7–9]. The plant, though often known as the hemorrhage plant or wild sunflower, is referred to by various names by different communities, such as Makayi in Luganda (Uganda), Orangila in Igbo (Nigeria), Nyana in Kissi (Sierra Leone), Fofo in Akan-akyem (Ghana), Mbnaso in Kpe (Cameroon), Soumadibrouin among the Malinke (Côte d'Ivoire), and Winnih in Mano (Liberia) [8, 10].

**Figure 1:**
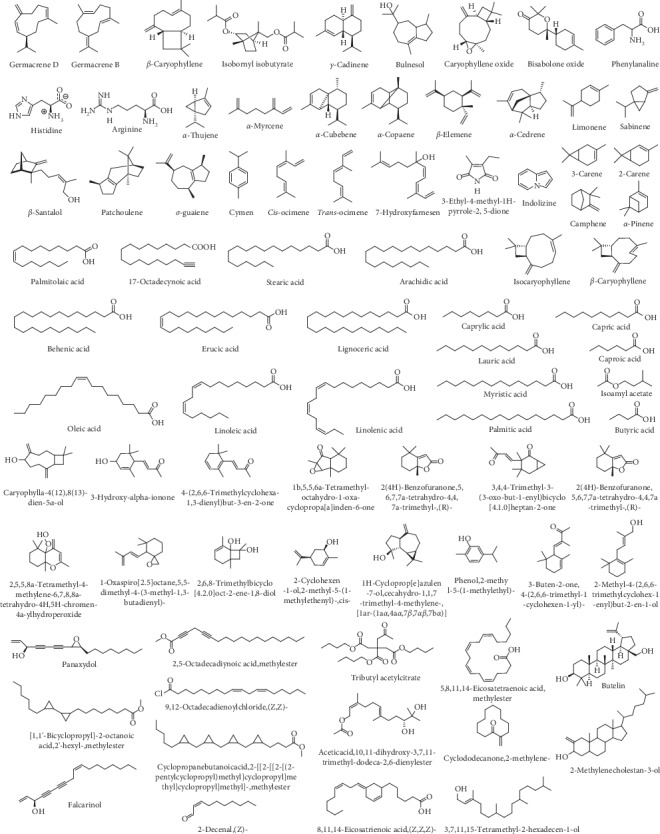
Molecular structures and names of different chemical compounds present in A. *africana* [9, 11, 12].

Analysis of the phytochemical composition of the plant indicates that the species is rich in a broad range of secondary metabolites, explaining its efficacy in treating inflammation-related diseases as well as other health conditions affecting different African communities (Figure 1). The therapeutic phytochemicals in *A*. *africana* include flavonoids, alkaloids, tannins, saponins, terpenoids, sterols, phenolic compounds, and glycosides (Table 1). Essential oils from the leaves of the plant are rich in monoterpenes, sesquiterpenes, *α*-pinene, and germacrene, which are important therapeutic ingredients [7]. The broad range of antimicrobial and biological activities, including anti-inflammatory, haemostatic, oxytocic, gastroprotective, antiulcer, wound healing, anticancer, antihypertensive, and antidiabetic potentials may be attributed to these groups of active therapeutic components of the plant [9, 19]. Typical secondary metabolites in *A*. *africana* that exhibit great anti-inflammatory potential include monoterpenes such as carene, terpenes including *α*-pinene, and sesquiterpenes such as *β*-caryophyllene and tannins among others (Table 2).

Most communities in developing countries rely heavily on traditional medicines, including herbal and medicinal flora and fauna, for their primary healthcare needs [8, 9, 23]. The World Health Organization (WHO) estimates that over 80% of the population worldwide relies on traditional medicine primarily for their healthcare needs [8, 23]. In a number of developed countries, herbal medicine is gaining popularity as alternative and complementary therapies due to their affordability, efficacy, and occurrence of fewer side effects compared to conventional drugs. According to Ahuchaogu et al. [9], the potential use of herbal plants as sources of new therapeutic drugs remains largely unexplored.

Currently used anti-inflammatory medications are associated with disadvantages such as drug toxicity, adverse effects, and iatrogenic reactions, thereby complicating the treatment process [5]. Dzoyem et al. [5] further stated that both steroidal and nonsteroidal anti-inflammatory drugs used in the treatment of severe inflammation have not been thoroughly effective, since most of these drugs increase the risk of blood clots and may result in stroke and heart attack. Some of these synthetic molecules, such as anticytokine agents, block the activity of several kinases and result in severe impairment of the immunity of an individual against infections [4]. The adverse effects related to the use of these conventional synthetic drugs have been the driving force behind consideration of natural remedies and the development of powerful anti-inflammatory drugs based on natural extracts. In this context, research should explore the properties of *A*. *africana* for the possible development of drugs, considering its long history of use as a traditional medicine for effective management of inflammatory diseases across Africa for centuries. The review is therefore important in that it provides valuable information on the ethnopharmacological potential of *A*. *africana*, thus offering a basis for extensive exploration of this important plant for developing new therapeutic anti-inflammatory drugs.

## 2. Materials and Methods

In this review, we modified the search process used in a previous study by Okello and Kang [24] to obtain information on *A*. *africana* from peer-reviewed articles published in scientific journals. The focus of the literature search was on anti-inflammatory activities along with other aspects of the plant, including its botany and ethnopharmacological or traditional medicinal uses in different communities across Africa. Electronic databases, including but not limited to PubMed, Scopus, Medline, Science Direct, SciFinder, and Google Scholar, were carefully explored for relevant information. The key words used for the search were as follows: (“*Aspilia africana*” OR “Hemorrhage plant” OR “Wild sunflower plant”) AND (“Botany” OR “Plant part structures” OR “Plant part morphology” OR “Plant part descriptions” OR “Medicinal uses” OR “Traditional medicinal uses” OR “Ethno-pharmacological uses” OR “Ethno medicinal uses” OR “Diseases treated” OR “Ethno medicine” OR “Pharmacological reports” OR “Wound healing properties” OR “Anti-inflammatory activities” OR “Anti-inflammatory potential” OR “Phytochemicals” OR “Phytochemical contents” OR “Phytochemical compounds” OR “Medicinal plant part used” OR “Plant parts used for treatment” OR “Herbal medicinal use”). The obtained information was verified independently for reliability, and any inconsistencies were settled through discussions among the authors. The information was analyzed, compared, summarized, and appropriate conclusions were drawn.

### 2.1. Morphological Features and Distribution of A. africana

**Figure 2:**
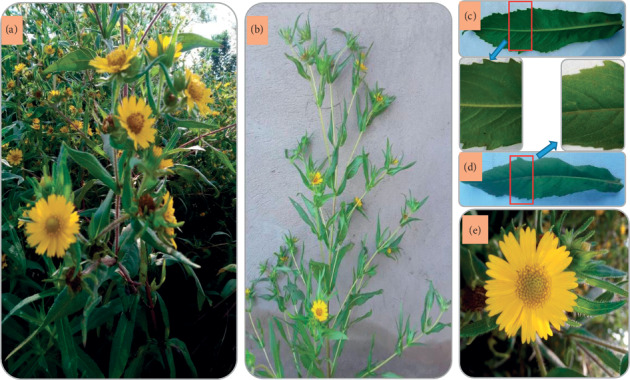
Botanical features of *A. africana* plant. (a) Plants growing in the wild, (b) stem characteristics and leaf arrangement, (c) features of lower leaf surface, (d) features of upper leaf surface, and (e) inflorescence.

*A. africana* is a perennial shrub that grows to a height between 60 cm and 2 m, depending on the availability of water and nutrition in the soil [2]. The stems are rough to touch, highly branched, and stiff from their bases (Figures 2(a) and 2(b)). The leaves are simple, oppositely arranged, ovate-lanceolate, creased accordion-style, and rough because the surfaces are covered with trichomes (Figures 2(b)–2(d)). The lamina have prominent midveins and are rounded at their bases but with pointed apices (Figures 2(c) and 2(d)); the leaves range from 6 to 25 cm in length and 3 to 7 cm in width and are attached to the plant stems by rough petioles measuring approximately 1 cm in length. Each hairy stalk of 4–10 cm length holds an inflorescence of solitary terminal capitulum. The inflorescence consists of numerous bright yellow florets that make the flowers conspicuous (Figure 2(e)). Bristly and finely hairy 4-angled achenes, approximately half a centimeter in length with diameters ranging from 0.5 to 2 mm, develop from each floret.

**Figure 3:**
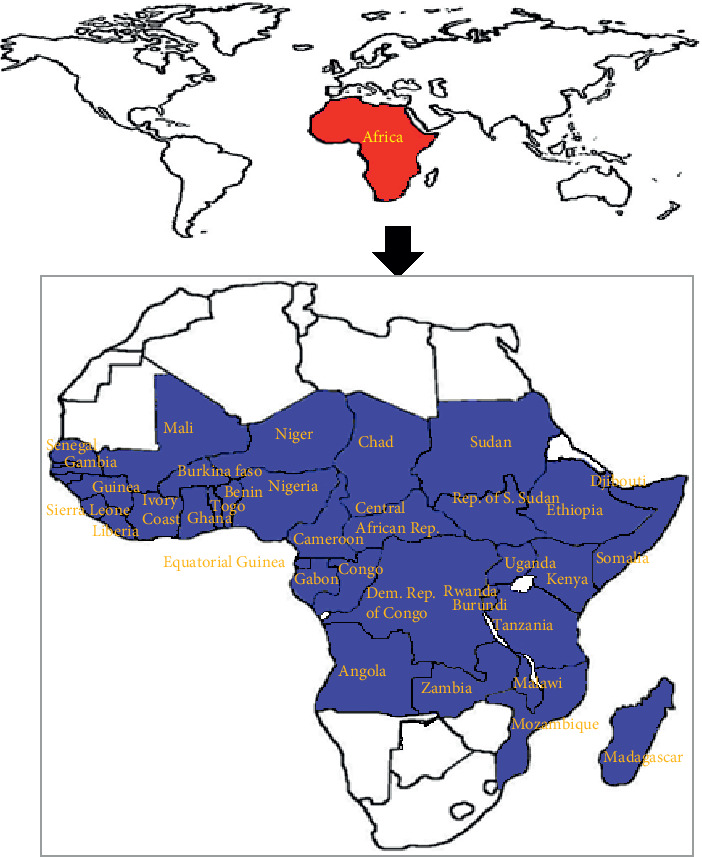
Tropical African countries with wide distribution of *A. africana* (modified from Komakech et al. [2].

*A*. *africana* is indigenous to East Africa, although it occurs in farmlands, wastelands, and forest zones in all regions of tropical Africa and the savanna [25]. The main areas of distribution of *A*. *africana* are the Eastern, Central, and Western African countries including Uganda, Tanzania, Congo, Sudan, Central African Republic, Benin, Cameroon, Burkina Faso, Chad, Gabon, Nigeria, Mali, Ethiopia, Guinea, Ghana, Liberia, Niger, Senegal, and Togo (Figure 3) [2, 25].

### 2.2. Anti-Inflammatory Properties of A. africana Extracts

*A. africana* extracts possess significant anti-inflammatory properties [2, 19]. These properties can be attributed to the presence of a wide range of secondary metabolites, including flavonoids, saponins, and tannins (Table 1). Several *in vitro* and *in vivo* studies have been carried out on *A*. *africana* extracts to determine their anti-inflammatory properties [2, 26]. *A*. *africana* fresh leaf extract is known for its wound healing potential, which is strongly linked to its anti-inflammatory activity that inhibits the synthesis of prostaglandins and neutrophil migration into the damaged tissues, reduces vascular permeability, and stimulates accumulation of lymphocytes, thus promoting the process of tissue repair [2, 19].

In a study that evaluated the systemic anti-inflammatory effect, hexane extract of *A*. *africana* significantly inhibited (*p* < 0.05) the progression of paw edema induced by the injection of egg albumin, with an activity greater than that of 100 mg/kg acetylsalicylic acid [19]. Okoli et al. [19] reported that acute inflammatory responses were suppressed by the hexane extract of *A*. *africana*. The anti-inflammatory effect of the extract could be due to inhibition of the release of and/or actions on a number of chemical mediators, including histamine, 5-hydroxytryptamine, prostanoids, and kinins, which play key roles in the mediation of acute inflammation induced by phlogistic agents [19]. Okoli et al. [19] further investigated and found that the hexane extract of *A*. *africana* effectively suppressed acute inflammation during the exudative phase. Hexane extract of *A*. *africana* significantly inhibited (*p* < 0.05) global edematous response to methanal-induced arthritis in a dose-dependent manner, producing an inhibitory effect greater than that of dexamethasone and an anti-inflammatory steroid [19]. Agnihortri et al. [1] also observed that the hexane extract of *A*. *africana* relieved rheumatic pain and suppressed the inflammatory response initiated due to tissue injury.

In another study, ethanolic extract of *A*. *africana* at a dose of 146.97 mg/kg prevented the formation of carrageenan-induced edema in rats, indicating significant (*p*=0.05) anti-inflammatory activity [27]. The study further stated that the extract also reduced paw size to 5.70 ± 0.16 mm from 6.75 ± 0.09 mm. The possible mechanism of action of the extract is inhibition of the action of serotonin and histamine or the synthesis of cyclooxygenase, since the inflammation caused by carrageenan is closely linked to the action of prostaglandins [26, 27]. Ilesanmi et al. [27] emphasized that the anti-inflammatory effect and mechanism of action of the ethanolic extract of *A. africana* were similar to those of nonsteroidal anti-inflammatory drugs, including ibuprofen, indomethacin, and aspirin.

In another study on the anti-inflammatory activity of *A. africana*, saline and ethanol extracts were found to be 85% and 90% effective, respectively, in stabilizing the membrane of bovine red blood cells against hypotonicity and heat lysis. This effect was comparable to that of a standard anti-inflammatory drug, indomethacin at 1 mg/ml [28]. Ukwueze et al. [26] investigated the anti-inflammatory effects of different extracts (n-hexane, dichloromethane, ethyl acetate, and butanol) and reported that for all the fractions, anti-inflammatory activities were not significantly different from those of indomethacin. However, significant positive differences (*p* < 0.001) were observed compared with the negative control group that used pure water extract.

### 2.3. Anti-Inflammatory Properties of Phytochemical Compounds in *A. africana*

Several studies have been conducted on the phytochemical compounds in *A*. *africana,* obtained from different regions of Africa, and significant variations in composition have been reported [13, 14]. Chemical analyses of the different plant parts indicate the presence of a wide group of therapeutically important classes of compounds, including flavonoids, alkaloids, tannins, saponins, terpenoids, sterols, phenolic compounds, and glycosides, most of which significantly contribute to the anti-inflammatory activities of the plant (Table 1) [2, 19, 26]. Aside from these secondary metabolites, individual phytochemical compounds in *A. africana* including carene, *a*-pinene, *β*-caryophyllene, and germacrene D (Table 2; Figure 1) are very important as far as the anti-inflammatory activity of this medicinal plant is concerned [19, 29, 30].

Flavonoids are a group of natural substances occurring in fruits, vegetables, grains, bark, roots, flowers, stems, wine, and tea with varying phenolic structures [31]. Flavonoids were known to be immensely beneficial to health, long before their isolation as effective therapeutic compounds [31]. There are over 4,000 flavonoid varieties, conferring bright colors to parts of plants, occurring as glycosides, methylated derivatives, and aglycones [31, 32]. It is therefore no surprise that they are abundantly present in *A. africana* flowers (Table 2). Flavonoids exist in several forms such as flavonols, flavanones, flavones, isoflavonoids, catechins, and anthocyanidins [31]. Ukwueze et al. [26] found that flavonoids were dominant phytochemicals in addition to being the main anti-inflammatory agent in *A*. *africana*. Under varying inflammatory conditions, flavonoids have been demonstrated to be phospholipase inhibitors, while some are tumor necrosis factor (TNF) inhibitors [26]. Depending on their structure, flavonoids are also known to be inhibitors of both lipoxygenase and cyclooxygenase (COX) pathways of arachidonic acid metabolism [33]. Komakech et al. [2] stated that flavonoids inhibit 5-lipoxygenase enzyme, thus playing an important role in suppressing leukotriene biosynthesis and reducing inflammatory reactions in the body. Rathee et al. [31] reported that flavonoids possess significant anti-inflammatory properties, despite their relatively unknown mechanism of action. The most predominant mechanism of anti-inflammatory activity of flavonoids is the inhibition of enzymes that generate eicosanoids such as phospholipase A2, lipoxygenases, and COX, thus causing a reduction in leukotriene and prostanoid concentrations. Other postulated mechanisms include inhibition of the release of histamine, protein kinases, phosphodiesterase, and transcriptase activation [31].

Saponins are a bioactive group of compounds present in abundance in *A*. *africana* (Table 1). Saponins derived from several plants demonstrated significant anti-inflammatory activity in a number of *in vivo* experiments on rat and mouse models [34]. Anti-inflammatory mechanisms of saponins include indirect and direct corticomimetic activity, inhibition of glucocorticoid degradation, inhibition of enzyme formation, and release of inflammatory mediators [34]. Tatiya et al. [35] observed that saponins obtained from the leaves of *Sesbania sesban* showed significant anti-inflammatory activity (*p* < 0.01) in the experimental models used, through the inhibition of rat paw edema induced by carrageenan and histamine. The results indicated that saponins play a crucial role in the acute inflammatory phase, with its action partly linked to histamine [35]. The significant ameliorative activity observed in the study could be a result of inhibition of inflammatory mediators by saponins, including histamine, prostaglandins, and serotonin [35]. In a different study, Mazumder et al. [36] indicated that crude saponins had a significant (*p* < 0.01) effect on granulomatous inflammation. The presence of large amounts of saponins in *A*. *africana* may play a significant role in its anti-inflammatory effects.

Tannins are a complex class of naturally occurring polyphenols, with most having the ability to precipitate gelatin and other proteins [37]. Tannins are classified primarily into three categories: condensed tannins (proanthocyanidins), hydrolyzable tannins, and phlorotannins [37]. Chemical analyses of different parts of *A. africana* indicated that the plant, especially the stem, is rich in tannins (Table 1). The anti-inflammatory mechanisms of tannins include free radical scavenging and inhibition of the expression of inflammatory mediators, including cytokines, COX-2, and inducible nitric oxide synthase (iNOS) [34]. A number of studies have indicated that tannins possibly affect inflammatory response through their free radical scavenging activities [38, 39]. Hydrolyzable tannins obtained from *Myricaria bracteata* had strong anti-inflammatory activity on ear edema induced by croton oil and arthritis in mice [40]. The study suggested that the anti-inflammatory action of tannins was linked to their potent antioxidant properties, and not their inhibitory effects on nitric oxide (NO) or on the reduction of proinflammatory cytokines [40]. In other studies, phlorotannins demonstrated powerful anti-inflammatory effects through the inhibition of cytokine release and production of prostaglandin-E2 and NO [41]. Terra et al. [38] observed that tannins reduced low-grade inflammatory diseases, including obesity, through the modulation of cytokine expression. Tannins are therapeutically important as anti-inflammatory agents and in the healing of wounds due to their astringent activities [42].

Alkaloids are a group of compounds that exhibit biodynamic activities in both humans and animals and possess one or more atoms of nitrogen in their heterocyclic ring [43]. Alkaloids are one of the largest classes of secondary metabolites in plants, and many studies have been conducted to investigate their anti-inflammatory activities [34, 43]. A number of alkaloids, including isoquinoline, diterpene, and indole, exhibit good anti-inflammatory activity [34]. Bisbenzylisoquinoline alkaloids such as isotetrandrine and cepharanthine have suppressive effects on the release of histamine and production of NO [34]. Cepharanthine further inhibited HIV replication in chronically infected monocytic cells, suppressed inflammatory cytokine production, and caused neural cell death [34]. Alkaloids in *Dendrobium crepidatum* inhibited the production of NO in lipopolysaccharide- (LPS-) activated macrophages, besides having protective effects on LPS-induced acute lung injury in mice [44]. A number of alkaloids possess significant anti-inflammatory properties, with some showing greater potential compared to aspirin [29]. The fact the *A. africana* contains a substantial amount of alkaloids, especially in the flowers (Table 1), could hint the potential use of this medicinal plant in the treatment of inflammatory diseases.

*β*-Caryophyllene, a sesquiterpene present in *A*. *africana*, is known to exhibit potent anti-inflammatory activity [2, 17]. *β*-Caryophyllene significantly reduced paw volumes (*p* < 0.01) and had low fluorescent signal intensity in mice [2]. Further, Komakech et al. [2] emphasized that the significant anti-inflammatory activities of *β*-caryophyllene, along with its remarkable antimicrobial activity, possibly explain the effectiveness of the plant in wound healing. Another study on the effect of *β*-caryophyllene in human monocytic cells (THP-1), through COX-2 activity assay, demonstrated powerful anti-inflammatory activity by COX-2 inhibition [22].

The terpenoid, *β*-pinene, in the leaves of *A*. *africana* possesses significant anti-inflammatory properties [2, 19]. The anti-inflammatory activity may be attributed to its potential to suppress mitogen-activated protein kinase (MAPK) and nuclear factor-kappa B (NF-*κ*B) pathways, making it an important compound in the treatment of inflammatory diseases [2, 45]. *α*-Pinene is also thought to contribute to wound healing by suppression of inflammatory reactions triggered by tissue injury [19, 30].

Carene is a monoterpene that exhibits strong anti-inflammatory activity [2, 21]. Carene demonstrated excellent dose-dependent anti-inflammatory activity against carrageenan-induced pedal edema [21]. Ocete et al. [21] further found that the anti-inflammatory activity of the essential oil extracted from *Bupleurum gibraltaricum* was chiefly due to the presence of carene. The mechanism of action of carene could be interference or inhibition of processes involving prostaglandins or their synthesis [21]. Carene being one of the chemical compounds in *A*. *africana* (Table 2) could be a contributing factor to its anti-inflammatory potential.

Another important chemical constituent in *A*. *africana* with significant anti-inflammatory potential is phytol, a diterpene alcohol [2, 20]). Phytol significantly inhibited paw edema development in mice (*p* < 0.05) induced by carrageenan dose dependently [20]. When 75 mg/kg of phytol was administered to the mice, 51.8% inhibition of paw edema development was recorded after four hours [20]. Further, phytol caused a significant reduction (*p* < 0.05) in the leukocyte recruitment and neutrophil migration to peritoneal cavity in mice [20]. Silva et al. [20] also observed that phytol significantly reduced interleukin-1*β* and TNF-*α* levels (*p* < 0.05) comparable to inhibitory effects produced by indomethacin. Thus, the anti-inflammatory effect exhibited by phytol is partly because it inhibits proinflammatory mediators and reduces migration of neutrophil and oxidative stress [20].

Linolenic acid, one of the chemical compounds present in *A*. *africana* (Table 2), is also a potent anti-inflammatory agent [2, 46]. It downregulates inflammatory iNOS, COX-2, and TNF-*α* gene expression by blocking NF-*κ*B and the activation of MAPKs in murine macrophage cell lines stimulated by LPS [2]. Reifen et al. [46] observed that *α*-linolenic acid effectively inhibited IL-1*β*-induced inflammation through downregulation of the levels of proinflammatory genes mRNA, which included COX-2 and IL-8. Thus, the presence of linolenic acid in *A*. *africana* potentially improves the plant's anti-inflammatory property.

A study conducted on germacrene D indicated that the compound exhibits potent anti-inflammatory activity [2]. Germacrene D, a sesquiterpene, is one of the phytochemicals in *A*. *africana* (Table 2). In a study of an essential oil extract from *Aster spathulifolius,* whose major component included germacrene D, dose-dependent suppression of the synthesis of both TNF-*α* and NO was observed, and it was thus recommended for cosmetic use owing to its anti-inflammatory effects [45]. In another study, an essential oil (LD_50_, 2.50 g/kg) with germacrene D as the main constituent inhibited writhing induced by acetic acid at a dose of 200 mg/kg in formalin test [47].

### 2.4. Toxicity Information on *A. africana*

*A. africana* is generally considered a valuable medicinal plant with low toxicity [2, 48]. The degree of toxicity of the plant leaf extract depended on factors such as exposure route, dose, and the extractant [25]. For ethanolic, aqueous, and chloroform leaf extracts of *A*. *africana*, the oral LD_50_ were 10,000 mg/kg, 12,589.3 mg/kg, and 1,995.3 mg/kg, respectively [25]. In mice, Oko et al. [25] discovered that behavioral toxicity signs included respiratory disorder, nervousness, and piloerection. In a study by Oluyemi et al. [49], intraperitoneal administration of *A*. *africana* leaf methanolic extract in Wistar rats caused damage to the uterine wall and significantly delayed their estrus cycles. In another *in vivo* study, oral administration of aqueous leaf extract of *A*. *africana* in female Wistar rats distorted the histology of their ovaries, impairing fertilization [30]. Further, *A*. *africana* aqueous leaf extract could be deforming to the developing placenta in Wistar rats in a dose-dependent manner [2]. A significant decrease in the weight of the testis, seminal vesicles, prostate gland, and epididymis was recorded in male Wistar rats on administration of the methanol extract of *A*. *africana* [50].

Oko et al. [25] noted that prolonged administration of *A*. *africana* plant products to animals prevented implantation and affected the activities of the reproductive system that were estrogen-dependent due to systemic hormonal imbalances, thus reducing fertility. Although Okwuonu et al. [51] demonstrated that *A*. *africana* methanolic extract did not induce weight loss in rats, Oyesola et al. [52] reported that weight and growth rate were significantly reduced in Wistar rats upon administration of leaf aqueous extract of the plant. Oyesola et al. [52] further noted reduced ovulation rate in a dose-dependent manner on administration of the aqueous extract of the plant, besides significantly causing fallopian tube inflammation, ovarian cortex degeneration, and uterine endometrial disruption.

Oko et al. [25] stated that the administration of an oral dose of up to 10,000 mg/kg body weight of *A*. *africana* leaf ethanolic and aqueous extracts was safe for animals and humans, thus supporting the common traditional use of the extracts as herbal medicinal remedies. Despite the effectiveness of *A*. *africana* in the treatment of a number of diseases, including those related to inflammation, it is important to use the extracts with caution. This is due to possible harmful effects, especially on the reproductive system, on prolonged use of high concentrations of the extracts.

## 3. Conclusion

Inflammatory diseases pose a global health challenge; thus, novel measures have to be considered to tackle the problem. Considering the challenges associated with the currently used anti-inflammatory drugs, it would be ideal to explore available natural remedies. *A*. *africana* is a plant that has been in use for centuries in the African continent for the treatment of inflammatory diseases and other health conditions; therefore, it could be considered a potential candidate in the development of newer anti-inflammatory therapies. As previously explained, several studies have investigated the anti-inflammatory activities of the extracts. In this review, we did not come across studies conducted on anti-inflammatory compounds isolated from *A*. *africana* itself but reported the results of studies on other plants that contained the same compounds. Nonetheless, *A*. *africana* is rich in secondary metabolites with significant anti-inflammatory potential, including flavonoids, alkaloids, tannins, saponins, terpenoids, sterols, and glycosides. Komakech et al. [2] pointed out that the synergistic effects of these phytochemicals in *A*. *africana* could be responsible for its anti-inflammatory potential. We therefore recommend future studies on anti-inflammatory activity based on phytochemical compounds isolated directly from *A*. *africana* plant parts, such as flowers and leaves. Based on the variety of secondary metabolites coupled with evidence from *in vitro* and *in vivo* studies regarding efficacy, *A*. *africana* could be more closely investigated for the potential development of novel anti-inflammatory therapeutics.

## Figures and Tables

**Table 1:** Profile of phytochemicals in different parts of *A*. *africana*.

Class of compound	Plant part	Type of extract	Status	Reference
Saponins	Stem	Ethanol (50%)	+++	[13]
	Flowers	Dry crude	+++	[14]

Tannins	Stem	Ethanol (50%)	+++	[13]
	Flowers	Aqueous	++	[14]

Flavonoids	Stem	Ethanol (50%)	++	[13]
	Flowers	Aqueous	+++	[14]

Alkaloid	Leaves	Aqueous	++	[15]
	Flowers	Dry crude	+++	[14]

Terpenoids	Leaves	Aqueous	+++	[16]
	Flowers	Chloroform	+	[17]

Steroids	Leaves	Aqueous	+	[15]
	Flowers	Dry crude	++	[14]

Phenolics	Leaves	Aqueous	++	[16]
	Flowers	Aqueous	+	[14]

Cardiac glycosides	Stem	Ethanol (50%)	+++	[13]
	Flowers	Aqueous	+	[14]

Anthraquinones	Stem	Ethanol (50%)	−	[13]
	Flowers	Dry crude	+++	[14]

Anthocyanins	Leaves	No data		
	Flowers	Aqueous	−	[14]

Carotenoids	Leaves	No data		
	Flowers	Aqueous	+++	[14]

Phlobatannins	Leaves	Chloroform	+	[18]
	Flowers	Dry crude	+	[14]

Key: +++ = abundantly present; ++ = moderately present; + = trace amount; − = absent.

**Table 2:** Selected key individual phytochemical compounds in A. *africana* with anti-inflammatory activities and their mechanisms of action.

Name of compound	Class of compounds	Chemical structure	Mechanism of anti-inflammatory action	References
*α*-Pinene	Terpenoid	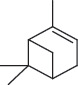	Suppression of MAPKs and NF-*κ*B pathway	[2]
Linolenic acid	Fatty acid	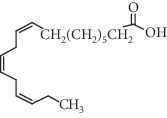	Downregulation of inflammatory iNOS, COX-2, and TNF-*α* gene expression	[2]
Phytol	Diterpene	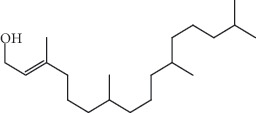	Inhibition of proinflammatory mediators and reduces migration of neutrophil and oxidative stress	[2, 20]
Carene	Monoterpene	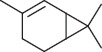	Inhibition of processes involving prostaglandins or their synthesis	[21]
*β*-Caryophyllene	Sesquiterpene	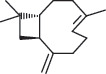	Inhibition of COX-2	[22]
Germacrene D	Sesquiterpene	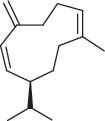	Suppression of synthesis of both TNF-*α* and NO	[15]
